# Functional Annotation of Rheumatoid Arthritis and Osteoarthritis Associated Genes by Integrative Genome-Wide Gene Expression Profiling Analysis

**DOI:** 10.1371/journal.pone.0085784

**Published:** 2014-02-14

**Authors:** Zhan-Chun Li, Jie Xiao, Jin-Liang Peng, Jian-Wei Chen, Tao Ma, Guang-Qi Cheng, Yu-Qi Dong, Wei-li Wang, Zu-De Liu

**Affiliations:** 1 Department of Orthopaedic Surgery, Ren Ji Hospital, School of Medicine, Shanghai Jiao Tong University, Shanghai, P. R. China; 2 Department of Anesthesiology, Ren Ji Hospital, School of Medicine, Shanghai Jiao Tong University, Shanghai, P. R. China; 3 School of Biomedical Engineering/MED-X Research Institute, Shanghai Jiao Tong University, Shanghai, P. R. China; Casey Eye Institute, United States of America

## Abstract

**Background:**

Rheumatoid arthritis (RA) and osteoarthritis (OA) are two major types of joint diseases that share multiple common symptoms. However, their pathological mechanism remains largely unknown. The aim of our study is to identify RA and OA related-genes and gain an insight into the underlying genetic basis of these diseases.

**Methods:**

We collected 11 whole genome-wide expression profiling datasets from RA and OA cohorts and performed a meta-analysis to comprehensively investigate their expression signatures. This method can avoid some pitfalls of single dataset analyses.

**Results and Conclusion:**

We found that several biological pathways (*i.e.,* the immunity, inflammation and apoptosis related pathways) are commonly involved in the development of both RA and OA. Whereas several other pathways (*i.e.,* vasopressin-related pathway, regulation of autophagy, endocytosis, calcium transport and endoplasmic reticulum stress related pathways) present significant difference between RA and OA. This study provides novel insights into the molecular mechanisms underlying this disease, thereby aiding the diagnosis and treatment of the disease.

## Introduction

Rheumatoid arthritis (RA) is a common chronic systemic autoimmune disease that mainly affects the flexible joints. It is characterized by the inflammation of articular synovial. The lasting recurrent inflammation of synovial can lead to the deformation and destruction of cartilage and bones, which could result in disability of the patients [1], [2]. RA mainly occurs in the 30∼70 years old people and is more frequent in females than males. More than 1% of the world’s population may be affected by RA [3], [4]. This disease brings great physiological and psychological burden to patients. However, the biological causes for RA remain largely unknown. Although infectious agents including viruses, bacteria and fungi have long been suspected, none has been comprehensively proved [5], [6]. Previous researches have also investigated the potential associations between RA and environmental factors, such as smoking, vitamin D deficiency, etc [7], [8]. It is now generally believed that the pathogenesis of RA is closely related to genetic factors. Certain genes such as the human leukocyte antigen (HLA). HLA-DR4 and DW4 antigen, were identified in more than 90% of the patients. These pathological factors are referred to as the RA-shared epitope [3], [9].

Osteoarthritis (OA) is another main type of chronic disease that affects the joints. The major pathological feature of OA is the degradation of articular cartilage and subchondral bone, and this may lead to the rigidity deformity and dysfunction of the joints [10]. The incidence of this disease in more than 50 years old people is as high as 80%. Etiological factors of OA include the mechanical injury, overweight, impairment of peripheral nerves, etc [11]. Osteoarthritis is different from rheumatoid arthritis in that there are extra-articular manifestations for rheumatoid arthritis. In addition, these diseases have different pathological manifestations for the synovial. RA is characterized by synovial cell hypertrophy and hyperplasia, infiltration of lymphocytes and inflammatory cells, whereas OA has fewer leukocytic infiltrates [12], [13].

Recently, large efforts have been made to screen the genetic factors involved in RA and OA by high-throughput methods [14], [15]. Several key genes and diagnostic markers have been identified for these diseases. However, the integrative analysis of multiple factors that contribute to the development of RA and OA appears to be a challenging task, and the underlying pathogenesis of RA and OA remain far from being understood. In this study, we compiled several whole-genome gene expression profiling datasets from RA and OA. Then we used a meta-analysis method to identify the aberrantly expressed genes. The subsequent functional annotation of these genes was performed based on gene ontology (GO) and Kyoto Encyclopedia of Genes and Genomes (KEGG) analysis [16], [17]. We demonstrated that several biological pathways are highly enriched in both RA and OA associated genes, such as chemokine signaling pathway, regulation of autophagy, focal adhesion, etc. Whereas other pathways, including regulation of autophagy, endocytosis, calcium transport and endoplasmic reticulum stress related pathways, are differentially influenced in the RA and OA respectively. This analysis provides a novel insight into the pathophysiological processes involved in these diseases. In addition, it would help to prioritize putative targets for further experimental studies and develop novel therapeutic strategies in preventing the RA and OA.

## Materials and Methods

### Sample Collection

**Figure 1:**
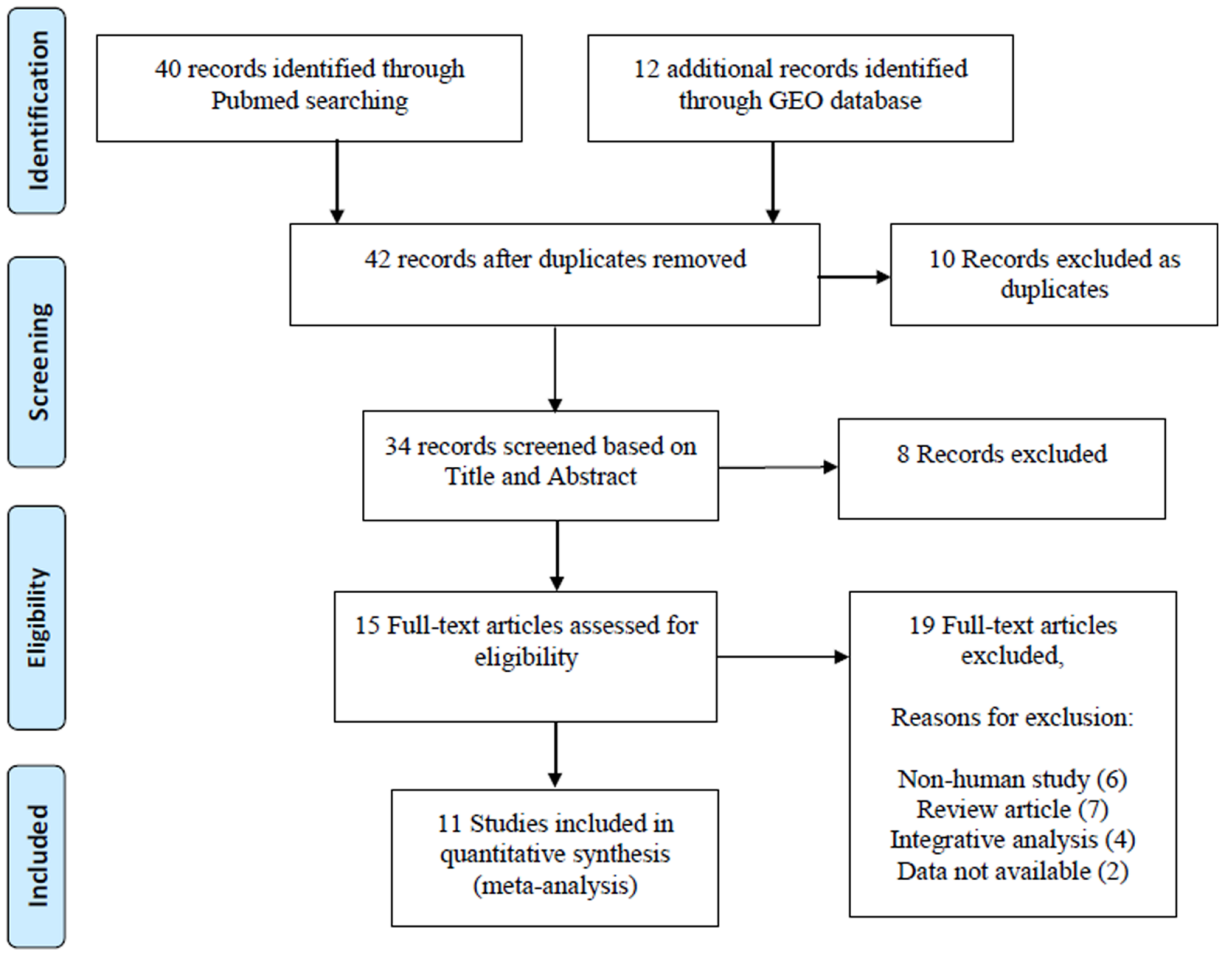
Flowchart of the selected process of microarray datasets for the meta-analysis.

We first queried the PubMed and related literatures to collect the expression profiling datasets from RA, OA and the corresponding normal control (NT) tissues. The following key words and their combinations were used: “rheumatoid arthritis, osteoarthritis, gene expression, microarray”. We only retained the original experimental works that analyzed the gene expression profiling between RA, OA and NT samples, respectively. Non-human studies, review articles and integrated analysis of expression profiles were excluded (**Figure 1**). At last, a total of 15 expression profiling datasets from 11 studies were collected (**Table 1**).

**Table 1:** Characteristics of analyzed datasets.

GEO Acc	PMID	Publish date	tissue type	Platforms	Number of samples
GSE1919	20858714	4-Nov-04	synovial	Affymetrix HGU95A	5 OA
					5 NC
					5 RA
GSE2053	20858714	10-Dec-04	synovial	HUMAN UNIGENE SetI Part 1	4 NC
					4 RA
GSE3698	16508983	6-Jun-06	synovial	Human Unigene3.1 cDNA Array 37.5K v1.0	18 RA
					19 OA
GSE7669	21474483	30-Aug-07	synovial	Affymetrix HGU95 2.0	6 RA
					6 OA
GSE9027	17665400	13-Sep-07	synovial		28 RA
GSE12021	18721452	2-Sep-08	synovial	Affymetrix HGU133A HGU133B Array	20 OA
					22 RA
					13 NC
GSE17755	21496236	21-Aug-10	peripheralblood	Hitachisoft AceGene Human OligoChip 30K 1 Chip Version	112 RA
					8 NC
					45 NC
GSE27390	21679443	31-May-11	bone marrow	Affymetrix HGU133 Plus 2.0	9 RA
					11 OA
GSE29746	22021863	25-Oct-11	synovial	Whole Human Genome Microarray 4x44K G4112F	9 RA
					11 OA
GSE36700	17469140	27-Mar-12	synovial	Affymetrix HGU133 Plus 2.0	6 OA
					7 RA
GSE39340		22-Oct-12	synovial	Illumina HumanHT-12 V4.0 expression beadchip	10 RA
					7 OA

A total of 11 expression profiles comparing RA, OA and NTs samples were collected in this study. Their GEO accession number, PubMed ID, publish date, tissue type, expression platform and number of samples were listed.

### Data Preprocessing

In this study, a global normalization method to minimize the data inconsistency and heterogeneity was used. We used the *Z-score* transformation approach to calculate the expression intensities for each probe of the gene expression profiles [18]. *Z-scores* were calculated according to the following formula:
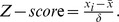
where x_i_ represents raw intensity data for each gene; 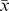 represents average gene intensity within a single experiment and 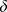 represents standard deviation (SD) of all of the measured intensities.

### Statistical Analysis

To give an overview of the global shifts of gene expression between pathological and normal tissues, we first calculated the pairwise Euclidean distances for samples from RA, OA and NTs according to the following the formula:
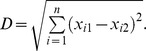



The significance analysis of microarray (SAM) algorithm was then used to identify the differentially expressed genes between pathological and control samples. The SAM procedure first calculate the “relative difference” score for each gene based on a modified *t-test* method, then a subsequent permutation analysis was used to compute false discovery rate (*FDR*) [19]. To get the best balance between the number of significant calls and the lowest *FDR* for the dataset tested, we used *FDR* <0.05 and |log fold change| >1 as the criteria for significant difference.

### Functional Annotation

In order to examine the biological significance of the differentially expressed genes, we performed GO and KEGG enrichment analysis to investigate their functional and pathway implications. The online based software GeneCoDis3 was used to perform this analysis [20]. The differentially expressed genes and all the expressed genes were submitted as the gene list and background list, respectively. The 5% cut-off of the FDR was used.

## Results

### Short Overview of the Studies Included

In recent years, many studies have used microarray technology to analyzed the whole genome expression proofing in samples of RA and OA. In this study, a total of 11 expression profiling datasets were collected, which include 383 samples. The characteristics of all these datasets included in this analysis were listed as **Table 1**. Among the 11 datasets, nine studies focused on synovial tissues and two studies focused on peripheral blood and bone marrow-derived mononuclear cells, respectively. More than half (six) of these studies focused on the differentially expressed genes between RA and OA samples, whereas four studies focused on the expression profiling of RA or OA and the corresponding NT samples, and one study only provided the expression profiling from RA samples.

### Global Changes in Gene Expression in RA and OA Samples

**Figure 2:**
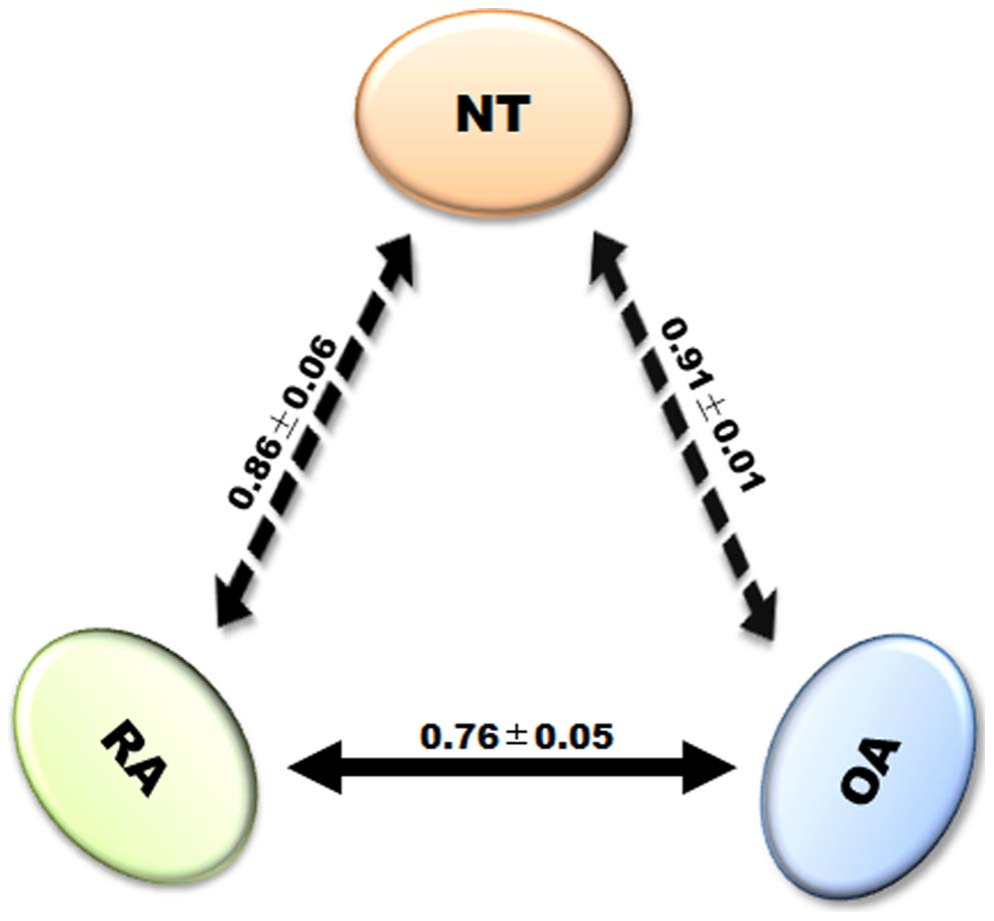
Average expression distances between RA, OA and NT samples. The average distances and the standard errors were labeled in the figure. The distance between pathological samples (RA or OA) and NTs is significantly larger than the distance between RA and OA samples.

Normalization is an important issue for comparison of microarray datasets. The heterogeneity of different datasets may lead to difficulties for comparing the results directly. The improperly normalized data used in microarray comparisons may run a high risk of skewing comparison results and reduces the credibility of individual gene change measurements. Towards this end, a global transformation of *z-score* was used to normalize all the expression profiling data retrieved for RA and OA. After filtering the normalized data, a total of 14,047 genes were detected in more than 60% of the samples. By using the assembled expression compendium, we investigated the global shifts of gene expression between RA, OA and NTs samples respectively. The average Euclidean distance was calculated to measure expression divergence between individual samples. As indicated in **Figure 2**, the expression divergence between OA and corresponding NT samples is significantly larger than that between RA and NT samples (Mann-Whitney U test, *P-value* <1e-6). We found that expression divergence between pathological samples and NTs is significantly larger than the distance between pathological samples of RA and OA samples (Mann-Whitney U test, *P-value*: 2.37e-57 and 1.12e-83 respectively,). This indicates that the similarities of expression signatures between RA and OA and several pathogenesis in common may contribute to the development of these diseases.

### Identification of Differentially Expressed Genes from RA *vs.* NT Samples

To obtain the genetic markers involved in the development and progression of RA and OA, the SAM method was used to identify the differentially expressed genes between pathological and control samples. At last, a total of 201 genes were found to be differentially expressed between RA and NT samples with the threshold of *FDR* <0.05 and minimal two-fold changes of expression. Among those differentially expressed genes, 35 genes were up-regulated and 166 genes were down-regulated in RA samples compared with the NT samples, respectively. The full list of these genes was provided in **Table S1**. The top 10 up-regulated and down-regulated genes for RA *vs.* NT were listed in **Table 2**, which include the *DCTN1, GABRR3, SOX18, ALPK2, UCP2, GGTL3, GNGT2, ABHD11, ETV3, NPCDR1,* etc. The gene with the most significant expression difference between RA and OA is Dynactin subunit 1 (*DCTN1*), which presents a ∼1437.67 fold higher expression in RA samples. *DCTN1,* encoding the largest subunit of dynactin, is involved in a diverse array of cellular functions, including the centripetal movement of lysosomes and endosomes, spindle formation, chromosome movement, nuclear positioning, and axonogenesis. Conversely, the gene with the most significant expression divergence and higher expression in RA samples (151.59 fold) is *NPCDR1.* Some of the deregulated genes have been previously reported to be closely related to the development of RA. For example, the single nucleotide polymorphism within the *UCP2* gene was identified to associate with many chronic inflammatory diseases including RA and systemic lupus erythematosus (SLE) [21]. Activated *PIAS1* gene was identified to repress the transcription of inflammatory genes [22], repression of *PIAS1* related pathways have some effects for the treatment of inflammatory disorders such as RA and atherosclerosis [23].

**Table 2:** Summary of differentially expressed genes between RA *vs.* NT samples.

Gene Symbol	Description	Score (D)	Fold Change	Status
DCTN1	dynactin 1	3.46	1437.67	down-regulated
GABRR3	gamma-aminobutyric acid (GABA) A receptor, rho 3	3.23	130.1	down-regulated
SOX18	SRY (sex determining region Y)-box 18	4.32	70.33	down-regulated
ALPK2	alpha-kinase 2	3.96	64.95	down-regulated
UCP2	uncoupling protein 2	3.85	52.75	down-regulated
MITD1	microtubule interacting and transport, domain containing 1	3.15	46.12	down-regulated
SMEK1	SMEK homolog 1, suppressor of mek1	3.64	35.59	down-regulated
BANF1	barrier to autointegration factor 1	3.46	33.62	down-regulated
PIAS1	protein inhibitor of activated STAT, 1	3.25	33.18	down-regulated
GFOD2	glucose-fructose oxidoreductase domain containing 2	3.38	30.77	down-regulated
TSPAN1	tetraspanin 1	−3.13	−48.9	up-regulated
HSPB2	heat shock 27kDa protein 2	−4.52	−50.67	up-regulated
NEK6	NIMA-related kinase 6	−10.5	−56.03	up-regulated
COL2	collagen, type II, alpha 1	−3.62	−62.02	up-regulated
LGALS9	lectin, galactoside-binding, soluble, 9	−4.85	−64.47	up-regulated
GGTL3	gamma-glutamyltransferase 7	−3.23	−79.05	up-regulated
GNGT2	guanine nucleotide binding protein (G protein), gammatransducing activity polypeptide 2	−9.81	−89.35	up-regulated
ABHD11	abhydrolase domain containing 11	−4.03	−95.86	up-regulated
ETV3	ets variant 3	−3.66	−106.96	up-regulated
NPCDR1	nasopharyngeal carcinoma, down-regulated 1	−4.79	−151.59	up-regulated

The symbol name, description, D score and the expression fold change were provided.

### Identification of Differentially Expressed Genes from OA *vs.* NT Samples

With the same analytic procedure described above, we identified 244 genes to be differentially expressed in OA samples comparing with NT samples, which include 45 up-regulated and 199 down-regulated genes. The full gene list and the top de-regulated gene list for OA *vs.* NT samples were listed in **Table S2** and **Table 3**, respectively. Specifically, the top deregulated genes for OA *vs.* NT samples include the *DPM2, MUS81, VAMP2, ZBTB33, NUP62, RHBDD1, PDZD7, PLEKHG4, ABCG1, TCEA3,* etc. Several of these genes have been identified to be involved in the development RA or OA samples, including *COL2* [24], *Gal-9* [25], [26], *MUS81* [27] and *ABCG1* [28].

**Table 3:** Summary of differentially expressed genes between OA *vs.* NT samples.

Gene Symbol	Description	Score (D)	Fold Change	Status
DPM2	dolichyl-phosphate mannosyltransferase polypeptide 2	4.18	697.61	down-regulated
MUS81	MUS81 structure-specific endonuclease	4.3	236.6	down-regulated
VAMP2	vesicle-associated membrane protein 2	6.38	220.45	down-regulated
ZBTB33	zinc finger and BTB domain containing 33	3.67	25366	down-regulated
NUP62	nucleoporin 62kDa	3.24	157.76	down-regulated
LOXL3	lysyl oxidase-like 3	4.84	111.57	down-regulated
LIMK2	LIM domain kinase 2	3.88	76.61	down-regulated
ZNF593	zinc finger protein 593	3.73	65.5	down-regulated
MRPS5	mitochondrial ribosomal protein S5	4.24	54.33	down-regulated
SMYD2	SET and MYND domain containing 2	3.62	31.41	down-regulated
MAN2	alpha Mannosidase II	−4.51	−87.45	up-regulated
SDCCAG1	NEMF nuclear export mediator factor	−6.12	−97.84	up-regulated
IGSF8	immunoglobulin superfamily, member 8	−6.9	−100.79	up-regulated
PBX1	pre-B-cell leukemia homeobox 1	−5.22	−133.75	up-regulated
KIAA1128	CCSER2 coiled-coil serine-rich protein 2	−6.9	−195.25	up-regulated
RHBDD1	rhomboid domain containing 1	−6.18	−231.9	up-regulated
PDZD7	PDZ domain containing 7	−4.48	−286	up-regulated
PLEKHG4	pleckstrin homology domain containing, family G(with RhoGef domain) member 4	−8.78	−337.12	up-regulated
ABCG1	ATP-binding cassette, sub-family G (WHITE), member 1	−3.87	−441.27	up-regulated
TCEA3	transcription elongation factor A (SII), 3	−8.37	−1241.14	up-regulated

The symbol name, description, D score and the expression fold change were provided.

### Functional Annotation of Differentially Expressed Genes

**Figure 3:**
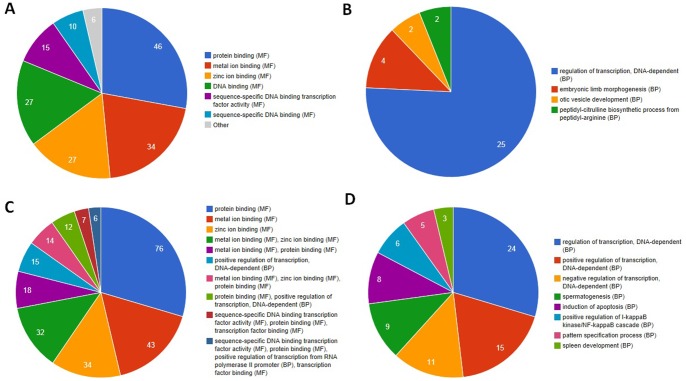
Enriched GO terms of differentially expressed genes between RA and OA *vs.* NT samples. (A) molecular functions for differentially expressed genes of RA *vs.* NT samples; (B) biological process for differentially expressed genes of RA *vs.* NT samples; (C) molecular functions for differentially expressed genes of OA *vs.* NT samples; (D) biological process for differentially expressed genes of OA *vs.* NT samples.

To gain insights into the biological roles of these differentially expressed genes from RA and OA *vs.* NT samples, we performed a GO categories enrichment analysis. GO category provides a descriptive framework of functional annotation and classification for gene sets analysis. GO categories are organized into three groups: biological process, molecular function and cellular component. In our work, only biological process and molecular function categories were considered. The functional enrichment work was performed by a web-based software, GeneCoDis3. With the *FDR* <0.05, we found GO terms for molecular functions significantly enriched in protein binding (GO:0005515), metal ion binding and DNA binding (GO:0003677), while for biological processes, the enriched GO terms were regulation of transcription (GO:0006355), embryonic limb morphogenesis (GO:0030326) and otic vesicle development (GO:0071599) (**Figure 3**).

To further evaluate the biological significance for the differentially expressed genes, we also performed the KEGG pathway enrichment analysis. The top enriched biological pathways associated with RA and OA include chemokine signaling pathway, glycosaminoglycan biosynthesis-chondroitin sulfate, SNARE interactions in vesicular transport, endocytosis, autophagy, etc. (**Table 4**). The chemokine pathway, for example, has long been suspected to involve in the development of RA. The chemokines are a family of small cytokines or signaling proteins secreted by cells, which function is to control cells of the immune system during processes of immune surveillance. Many chemokines could participates in the inflammatory response and attracts immune cells to the site of inflammation [29]. The genes involved in chemokines signaling pathway was identified to be altered in both RA *vs.* NT and OA *vs.* NT. The *CXCL2*, for example, also known as *GRO2*, is implicated in the recruitment of neutrophils from the circulation system to the sites of inflammation [30], it is constitutively expressed in resting OA cells, which supports the idea that some circumstances OA can be considered inflammatory disease [31].

**Table 4:** KEGG pathway enrichment of genes differentially expressed RA and OA *vs.* NT samples.

Sample type	KEGG pathway	Number of genes	Entrez gene ID	*P-value*
OA vs. NT	Chemokine signaling pathway	5	2920 2869 409 9844 2309	0.0168
OA vs. NT	Endocrine and other factor-regulated calcium reabsorption	5	56302 6546 490 793 8766	0.0179
OA vs. NT	Glycosaminoglycan biosynthesis - chondroitin sulfate	3	55501 64132 10090	0.0286
OA vs. NT	SNARE interactions in vesicular transport	3	6844 53407 113189	0.0462
OA vs. NT	NF-KAPPA B signaling	5	27040 29760 8915 10015 2637	0.0439
OA vs. NT	PPAR signaling pathway	3	1376 364 10999	0.0726
OA vs. NT	Protein processing in endoplasmic reticulum	4	50613 9695 3300 4217	0.0726
OA vs. NT	Phagosome	3	53407 23673 30835	0.0726
OA vs. NT	Pathogenic Escherichia coli infection	3	9181 999 10092	0.0726
OA vs. NT	MAPK signaling pathway	3	999 9844 6197	0.0726
OA vs. NT	Focal adhesion	3	6844 3783 3912	0.0974
OA vs. NT	Oxidative phosphorylation	4	9997 4519 28487 4512	0.0981
OA vs. NT	Endocytosis	3	2869 23096 409	0.0996
RA vs. NT	Vasopressin-regulated water reabsorption	3	6844 51164 1639	0.0175
RA vs. NT	Glyoxylate and dicarboxylate metabolism	3	4190 48 847	0.0275
RA vs. NT	Chemokine signaling pathway	3	2885 2793 2829	0.0493
RA vs. NT	Regulation of autophagy	3	25989 5562 9474	0.0339
RA vs. NT	NF-KAPPA B signaling	4	29760 8915 8091 9020	0.0339
RA vs. NT	SNARE interactions in vesicular transport	3	6844 9527 9482	0.0339
RA vs. NT	Oxidative phosphorylation	5	4508 9997 4519 28487 4512	0.0421
RA vs. NT	Citrate cycle (TCA cycle)	3	4190 48 945406	0.0421
RA vs. NT	mTOR signaling pathway	3	25989 5562 1978	0.0421
RA vs. NT	Adherens junction	3	81 6615 7414	0.0781
RA vs. NT	Wnt signaling pathway	4	56998 6093 9475 51176	0.0781
RA vs. NT	MAPK signaling pathway	4	9448 4773 7151 4217	0.0793
RA vs. NT	Pathogenic Escherichia coli infection	3	9181 999 4690	0.0793
RA vs. NT	Focal adhesion	3	81 2885 3912	0.0841
RA vs. NT	Bacterial invasion of epithelial cells	3	10163 999 9844	0.0841

The number of differentially expressed genes in a specific pathway, enriched gene ID and adjusted *P-values* calculated by fisher’s exact test were included.

As for apoptosis, genes involved in the regulation of cell survival and anti-apoptosis and autophagy related pathways are significantly affected, such as the mitogen-activated protein kinases (MAPK) pathways. MAPK comprises a family of serine/threonine protein kinases that implicated in the regulation of key cellular processes including cell survival, proliferation, differentiation and apoptosis as well as cellular stress and inflammatory responses. The respective genes in MAPK pathway showed altered expression levels in RA and OA patients of this study. Involvement of MAPK in the regulation of the synthesis of inflammation mediators and the development of RA have been widely identified [32]. Inhibitors targeting MAPK related pathways have been developed and the preclinical data indicated that they exhibit anti-inflammatory activities. This makes them the potential targets of anti-inflammatory therapy for these diseases [33].

Focal adhesion kinase (FAK) is another pathway that known to play a key role in cell proliferation and migration. Members of this family, which include FAK and PYK2 and their associated signaling intermediates, have been implicated in cell adhesion, migration and osteoclast differentiation [34]. GRB2 is one of the interaction factors of FAK that facilitate intracellular signaling [35]. This gene was found to be up-regulated in both of the diseases, and this may be responsible for the activation of the FAK family signaling and results in the adhesion and migration of the pathological cells.

## Discussion

Rheumatoid arthritis and osteoarthritis are the most commonly observed types of arthritis. However, the underlying causes of RA and OA remain largely unknown. Understanding the pathogenesis could have important implications for drug development and treatment for these diseases. Genetic researches on RA and OA have pursued throughout the last years. For example, the whole genome expression profiling studies by using microarrays. Gene expression profiling studies are capable of identifying differences in transcription of thousands of genes on a genome-wide scale. This technique may investigate the pathophysiology of complex genetic tracts and the altered molecular pathways. The first genome wide comparisons of gene expression of RA and OA was performed by Ungethuem *et al.* in 2006. To date, a total of 11 microarray mRNA profiling studies comparing RA and OA with control tissue have been published. Combination and comparison of these studies may have the potential to substantiate and filter the results of each single study and may provide further insights into the pathogenesis of these diseases. However, the heterogeneity of the datasets may run a risk of skewing comparison results and reduces the credibility of gene expression change measurements. To this end, we collected those published expression profiling datasets and used a global normalization method to calculate the expression level for each gene. This algorithm used in this study could reduce heterogeneity of different datasets and make them comparable. Then we performed a systemic meta-analysis based on re-analysis of primary data sets to retrieve RA and OA associated genes. Followed functional implication analysis was performed to investigate their physiological impact in development of these diseases. To our knowledge, no other systematic meta-analysis of gene profiling has been performed to investigate the differences and similarities between RA and OA. The present study suggests several promising genes and may provide a clue to the role of these genes played in the development of these diseases.

Based on our results, it is evident that inflammation as well as apoptotic processes are key elements in the development and progression of RA since several inflammation- and apoptosis- associated genes were identified. For example, the *Gal-9*. *Gal-9* is a kind of immunity associated gene that plays a role in inflammatory responses. This gene has previously been proved to be a ligand of T cell Ig and mucin domain (*Tim-3*). It was reported that *Tim-3* expression is higher in patients with inflammatory disorders such as RA compared to controls [36]. In this case, up-regulation of *Gal-9* may enhance the *Tim-3-Tim-3L* interactions in synovial and improve the symptoms of inflammation.

In addition, we found that the NF-κB signaling pathway may play significant role in these diseases. NF-κB is a key transcription factor that regulates a variety of genes involved in immune response, cell differentiation and proliferation. Incorrect activation of NF-κB was suggested to associate with cancer, inflammatory and autoimmune diseases, septic shock and viral infection [37]. It has previously indicated that Interleukin-1 beta (*IL-1β*) gene was induced in the RA patient-derived synovial fibroblast cell line MH7A by cigarette smoke condensate [38]. NF-κB binding sites were found in the promoter region of *IL-1β* gene. Therefore, this indicated that aberrant expression of the genes relevant of NF-κB signaling pathway may play a pathological role in the development of RA and OA.

Abnormalities in the mitochondria have been a topic of interests into the study of arthritis. It has been reported that mutation frequency of mtDNA is significantly higher in the inflamed synovial compared with normal synovial. This high mutation frequency is caused by the inflammatory mediators of TNFα and interferon γ (IFNγ) and eventually results in the changes of microenvironment and function of mitochondria [39]. Here, we reported that expressions of certain genes related to the function of mitochondria were altered in RA patients. Notably, the relevant genes, which include the *ATP6, SCO2*, *CYTB, DN1, COX1, ANT1*, are mainly function in oxidative phosphorylation, whereas dysfunction in oxidative phosphorylation related genes is closely related to the systemic juvenile idiopathic arthritis and endemic osteoarthritis [40], [41]. This largely indicates that both RA and OA can be classified as mitochondrial disorder.

Although RA and OA samples share many similarities of their respective gene expression profiles and a number of pathways show comparable variance in both of these diseases, thus reflecting basic common pathomechanism of these joint diseases. However, RA and OA samples can be clearly differentiated regarding gene expression variances in other pathways. In OA, the pathways affected by expression variances include calcium ion transport, PPAR signaling pathway, protein processing in endoplasmic reticulum (ER), phagosome and endocytosis related pathways, etc. Calcium is the essential structural component of the skeletal system. Adequate calcium intake is the basis of osteoblast growth. Observation of the dysregulated expression of calcium related gene may partially explain why calcium pyrophosphate dihydrates accumulate in synovial of OA patients [42], [43]. Endocytosis and autophagy are the major pathways for materials to be transported into the lysosomes in cells. The former is responsible for uptake of extracellular constituents and the latter for degradation of cytoplasmic constituents. Several common factors and pathways that regulate the endocytosis and autophagy has been identified [44]. Since there is a high correlation between autophagy activation and the severity of experimental osteoarthritis [45], we may speculate the causal relationship between the deregulation of endocytosis related genes and the development of OA.

ER stress refers to as the enhanced expression of normal or folding-defective proteins and the accumulation of unfolded protein in ER by stimuli. This process has been shown to participate in many disease, including diabetes, inflammation, and neurodegenerative disorders [46]. It was also indicated that ER stress may contribute to chondrocyte apoptosis along with OA progression, which was closely associated with an enhanced apoptotic response and a reduced protective response by cells [47]. Therefore, molecules that regulate the ER stress response would be candidate targets for treatment of this disease.

In contrast to OA, RA-specific pathways are involved in vasopressin-regulated water reabsorption, adherens junction, etc. As a proinflammatory hormone, vasopressin can stimulate the cell proliferation in chondrocytes that derived from patients with RA [48]. Adherens junctions are protein complexes that occur at cell-cell junctions in epithelial tissues to create ephemeral connections with counterparts from adjacent cells. The inflamed synovial tissue undergoes remodeling during the course of RA, the synovial lining becomes hyperplastic and forms a condensed tissue [49]. Genes related to the adherens junctions pathway is speculated to involve in this process and their abnormal expression may enhance the development of RA. Identification of interferon signaling and bacterial invasion related pathways suggests that some of the cases are indeed caused by microbial infection. In addition, other canonical pathways that involved in the RA development, such as the Wnt signaling and mTOR signaling pathways, were also identified in this analysis [50], [51]. These affected pathways and the respective genes reported here may provide the basis for further analyses of the pathogenesis and the differences between RA and OA on a cellular and molecular level.

In conclusion, by collecting the whole genome expression data sets from different platforms, multiple biological markers were identified for RA and OA. This work is important to characterize the specific roles of those genes involved in the pathogenesis of RA and OA. Functional analysis of these genes may provide additional insights into the complex process of these diseases. In addition, this analysis may help to improve the diagnosis and treatment of these diseases.

## Supporting Information

Table S1 **Full lists of the differentially expressed genes between RA** ***vs.*** **NT samples.** The symbol name, D score and the expression fold change were provided.
(XLSX)

Table S2 **Full lists of the differentially expressed genes between OA** ***vs.*** **NT samples.** The symbol name, D score and the expression fold change were provided.
(XLSX)
